# Design and Dynamic Control: A Free-Flying Space Robot Inspired by Water Striders

**DOI:** 10.3390/biomimetics8050437

**Published:** 2023-09-19

**Authors:** Huayang Sai, Chengkai Xia, Zhenbang Xu, Hang Li

**Affiliations:** 1 College of Engineering, Peking University, Beijing 100871, China; saihuayang@pku.edu.cn; 2 CAS Key Laboratory of On-Orbit Manufacturing and Integration for Space Optics System, Changchun Institute of Optics, Fine Mechanics and Physics, Chinese Academy of Sciences, Changchun 130033, China; xiachengkai19@mails.ucas.ac.cn (C.X.); lihang@ciomp.ac.cn (H.L.); 3 University of Chinese Academy of Sciences, Beijing 100049, China

**Keywords:** free-flying space robot, sliding mode control, fuzzy control, on-orbit assembly

## Abstract

This work designed a free-flying space robot (FFSR) that simulates the on-orbit assembly of large space telescopes, drawing inspiration from the flexible movement of water striders on water surfaces. Initially, we developed the system structure of the robot, including the corresponding air-floating ground simulation system. This system enables floating movement of the robot in a gravity-free environment through the utilization of planar air bearings. Subsequently, we established the kinematics and dynamics models for the FFSR. Following that, we propose a novel adaptive boundary layer fuzzy sliding mode control (ABLFSMC) method to achieve trajectory tracking control of the FFSR. The virtual angle and angular velocity are formulated to serve as references for the angle and angular velocity in the body coordinate system. Furthermore, a fuzzy logic system is employed to minimize the chattering effect of the sliding mode control. The global stability of the proposed controller is guaranteed through the Lyapunov stability theory. Finally, we validate the effectiveness of the proposed control method as well as the high trajectory tracking accuracy of the developed FFSR through simulation and experimental results, respectively. Overall, our findings present a crucial experimental platform and development opportunity for the ground-based validation of technologies concerning the on-orbit assembly of large space telescopes.

## 1. Introduction

The development and deployment of large-aperture space telescopes have become a significant research area due to the crucial role played by large space optical telescopes (LSOTs) in space exploration missions, which is particularly relevant with the growing need for deep space exploration [1,2,3]. Nonetheless, the expanding aperture of the telescope is greatly limited by the carrying capacity of rockets. In-orbit assembly technology, based on the concept of LSOT segmented assembly, emerges as a highly promising and effective approach for constructing future LSOTs [4,5,6,7,8].

Free-flying space robots (FFSRs) possess the capability to revolutionize space exploration by enabling a variety of on-orbit operations. In recent years, numerous scientific research institutions have been contemplating the utilization of robotic systems in space missions, particularly within the context of large-scale space equipment assembly [9,10]. For instance, the Surrey Space Center has proposed the concept of autonomously assembling a reconfigurable space telescope through the utilization of microsatellite and docking technology in orbit [11]. Additionally, the MIT Space Systems Laboratory put forth the idea of assembling space telescopes by employing multiple satellites in orbit [12]. The China Academy of Space Technology presented an integrated assembly scheme that utilizes space robots to actualize the concept of a Space Solar Power Station [13]. However, technologies associated with FFSRs pose significant challenges, and conducting ground-based experiments remains the sole approach for their development. Currently, certain researchers have explored various distributed air-bearing simulation systems with three degrees of freedom (DOFs) to simulate spacecraft position and attitude motion separately [14,15,16]. There is still substantial work to be conducted in systematically simulating FFSRs [17,18,19]. To fulfill the demands of on-orbit simulations, which include docking, rendezvous, and formation flight, it is crucial to develop FFSRs capable of accurately tracking trajectories for algorithm validation and to serve as a platform for validating technologies associated with space on-orbit assembly. It is worth noting that the majority of existing FFSRs primarily focus on verifying space formation flight, rendezvous, and docking missions [20,21,22,23,24,25]. They are typically designed with smaller dimensions and flexible mobility. However, these FFSRs often suffer from limited carrying capacity and shorter endurance, which hinders their suitability for space telescope ground simulation assembly missions.

When utilized in ground-based simulations, FFSRs necessitate the precise tracking of relative position and synchronization of attitude between the robots and targets, particularly when dealing with non-cooperative targets [26]. While striving for accurate trajectory tracking and attitude synchronization, it is essential to address both the coupling and nonlinearity of the robot system as well as ensure the control system exhibits robustness. To address the issue of nonlinear coupling in the control of FFSRs, several methodologies have been attempted, including feedback linearization [27], model predictive control [28], and sliding mode control (SMC) [29]. Among these control algorithms, SMC demonstrates excellent performance in mitigating external disturbances and nonlinear factors, which has led to its widespread utilization in robotics [30,31], aerospace [32,33], and servo systems [34,35]. For example, in [34,35], researchers utilized extended sliding-mode observers to simultaneously estimate the system’s inertia and load torque, which benefits from its high accuracy and fast convergence. Nevertheless, the classical sliding mode control suffers from an inherent drawback known as chattering. To eliminate chattering, numerous methods have been put forward by researchers. One simple and easy-to-implement method involves surrounding the sliding surface with a boundary layer, allowing the relay control to be restored using a saturation function block [36]. Researchers utilize fuzzy control and SMC together to mitigate the effects of chattering without compromising system robustness [37]. The fuzzy rule is employed to adjust the control gain of the SMCer constructed by the approach law, effectively reducing the chattering phenomenon. Yang et al. [38] utilized the SMCer on unmanned quadrotors and introduced an adaptive fuzzy gain-scheduling algorithm. This algorithm employs fuzzy control to adjust specific parameters of the approach law, thereby enhancing the response speed of the control system. Gao et al. [15] devised a position and attitude controller utilizing fuzzy SMC and demonstrated its efficacy. However, when establishing the dynamic model, Gao et al. [15] only considered the dynamic relationship in the body coordinate system, and there are too many coefficients which are set in the control system. In contrast to the elaborate construction of complex dynamical models, fuzzy control can be applied without requiring prior knowledge of the system model [39], which makes it an ideal approach for nonlinear control tasks that involve uncertain input and output parameters.

This paper presents the design of an FFSR for validating on-orbit assembly technology for space telescopes, drawing inspiration from the agile locomotion of water striders on water surfaces. The design of this FFSR takes inspiration from the flexible movement exhibited by water striders on the water surface. With its high load capacity and long endurance, the designed FFSR serves as an ideal platform for validating ground-based simulations of space telescope on-orbit assembly. To improve the tracking accuracy of the robot in trajectory tracking and attitude synchronization, a novel adaptive boundary fuzzy sliding mode control (ABFLSMC) method is proposed for trajectory tracking and attitude tuning of FFSR, including a position and an attitude controller. For the attitude controller, a double-closed-loop control method is employed to address the coupling between kinematics and dynamics, thereby circumventing the need for complex transformations of the Euler angle and angular velocity in the body coordinate system. The outer loop implements the integral SMC method for Euler angle tracking, while the inner loop achieves tracking of the virtual reference angular velocity, which solves the problem that the robotic attitude dynamics cannot be directly applied. Simulations and experiments are conducted respectively to demonstrate the effectiveness of the proposed control method and the designed FFSR in performing the trajectory tracking task.

The rest of the paper is organized as follows. The structure of the FFSR system and the principle of movement of its position and attitude are described in Section 2. According to the robotic physical model, the Newton–Euler method is used to establish the dynamics model in Section 3. In Section 4, the ABFLSMC method is described based on the established dynamic model, and the definition of the fuzzy rule is discussed. The control system is simulated and experimentally evaluated in Section 5. Finally, conclusions are drawn in Section 6.

## 2. Design of the Free-Flying Space Robot

### 2.1. Mechanical Design of the FFSR

**Figure 1:**
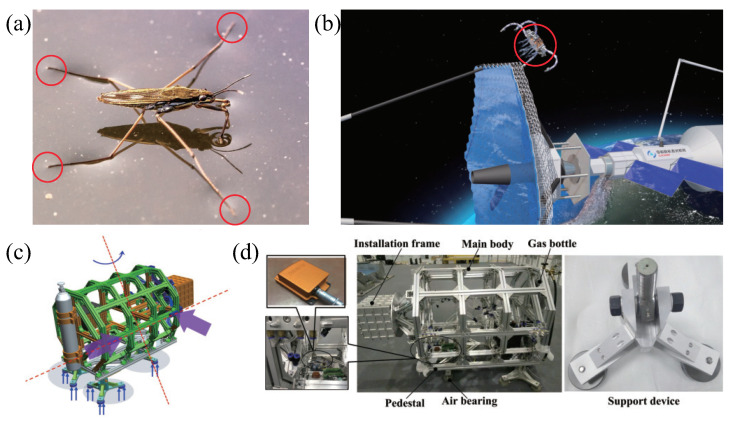
Mechanical design of the FFSR. (**a**) Water striders moving on the surface of the water. (**b**) Hypothetical view of LSOT assembly in orbit. (**c**) Schematic of the movement of the FFSR. (**d**) Mechanical design and detail drawing of FFSR.

Nature-inspired structural design has become increasingly popular among scientists and engineers in recent decades. Certain semi-aquatic arthropods, such as water striders, have developed the capacity to float and navigate on the water’s surface, as depicted in Figure 1a. This serves as a source of inspiration for the advancement of free-flying space robots, as shown in Figure 1b, with the objective of executing the LSOT on-orbit assembly mission. The limbs of the water strider make contact with the water surface, creating small pits that generate capillary forces, supporting its ability to float. To establish a simulation system on the ground and to draw inspiration from this phenomenon, we designed four sets of aerostatic bearings for the FFSR. These bearings are intended to form a thin film of air with the ground, enabling levitation, as shown in Figure 1c. The developed FFSR achieves levitation through the use of planar air bearings and accomplishes propulsion and steering movements by employing air nozzles. The designed FFSR incorporates four suspension supports at its base, and each support features three aerostatic bearings for suspension and side-mounted nozzles for movement, as demonstrated in Figure 1d. The main structure of the FFSR is designed with trusses, effectively reducing the overall mass. An external interface is incorporated on each side, allowing for the easy expansion of the robot’s functionality. Moreover, the FFSR is equipped with an inertial measurement unit that enables the real-time detection of attitude angle, angular velocity, three-axis motion velocity, and acceleration. These measurements are crucial for closed-loop tracking control.

Figure 1d depicts the principal components of the FFSR, encompassing the main truss, the base, and the robotic arm support. The pedestal serves as a mounting point for the air bearing, which can be detached from the main truss. The aerostatic bearing is connected to the base through three supporting devices, which are themselves linked to the base using rod end-joint bearings. The FFSR incorporates a cold air propulsion system with liquid carbon dioxide serving as the gas source. Analysis of the factors influencing nozzle thrust reveals that there is minimal thrust increase as the pressure continues to rise beyond 0.4 MPa. However, higher pressure levels lead to elevated gas consumption. Therefore, the system is configured to operate at a working pressure of 0.4 MPa. Additionally, the physical layout clearly demonstrates a symmetrical arrangement of the nozzles. Previous experimental test results have confirmed that each individual nozzle generates a thrust of 1.025 N under the specified working pressure [40]. The structural parameters and performance indexes of the designed FFSR are shown in Table 1, which can meet the mission requirements.

### 2.2. Drive System Design of FFSR

**Figure 2:**
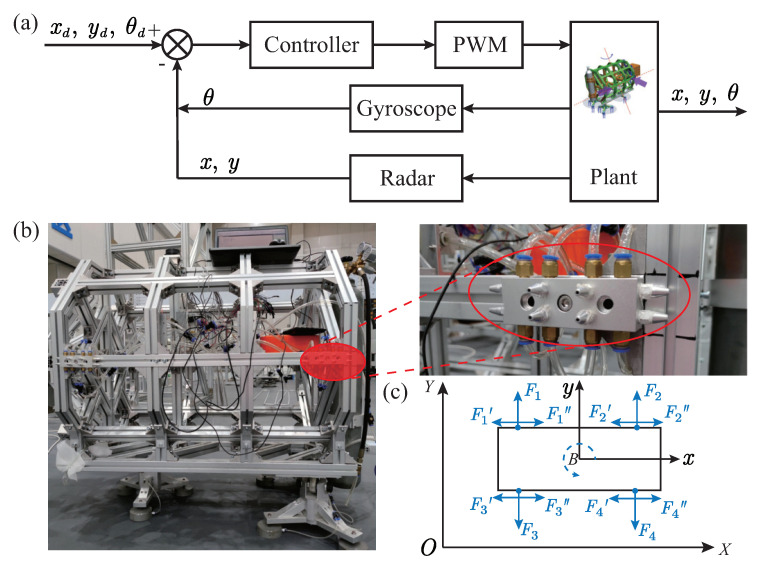
Drive system design of FFSR. (**a**) Diagram of the control principle of FFSR. (**b**) Schematic diagram of the gas nozzle. (**c**) Cold gas propulsion system.

**Figure 3:**
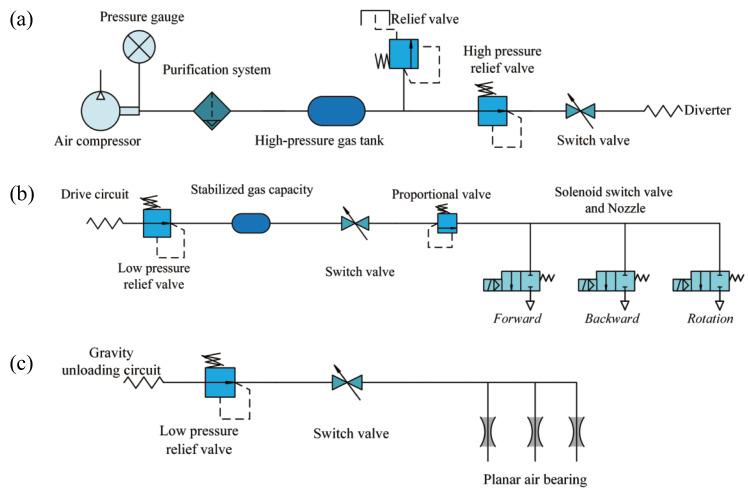
The principal design drawing of the gas circuit of the FFSR. (**a**) Main air circuit. (**b**) Drive circuit. (**c**) Gravity unloading circuit.

The control system of the developed FFSR is illustrated in Figure 2. The controller receives the target position and desired attitude angle; then, it calculates the thrust required for the thruster. This thrust drives the FFSR to perform planar motion. To ensure accurate tracking, the controller utilizes feedback from the position and attitude angle by incorporating a gyroscope and LIDAR into the control system. This allows for information processing and establishes a closed-loop control of the system. The gyroscope, functioning as an inertial navigation sensor, not only captures the angular velocity increment during FFSR movement but also generates attitude angle information. The LiDAR scans the 2D plane 360${}^{\circ }$ to gather distance information of the target within the surrounding environment. Through coordinate transformation, the corresponding position information can be obtained. To accurately simulate the motion of the FFSR in space, a cold gas propulsion system is employed. The FFSR is designed with symmetrical arrangements of four groups of gas nozzles on each side, allowing for independent gas injection along three directions, as depicted in Figure 3b. Consequently, the cold gas propulsion system enables planar motions in three translational dimensions, namely the x-axis and y-axis, as well as rotational motion around the z-axis, as illustrated in Figure 3c.

### 2.3. Gas Circuit Design of the FFSR

The gas path of the FFSR comprises two components: cold gas propulsion and planar aerostatic bearing. The gas circuit system of the entire device is illustrated in Figure 3. The high-pressure carbon dioxide gas undergoes depressurization through a two-stage pressure reducing circuit, effectively adjusting the air pressure within the desired range while ensuring consistent nozzle thrust output. Subsequently, the gas is filtered to remove moisture and oil mist using a filter valve, ensuring the cleanliness of the gas. A gas cylinder is installed in the propulsion branch to supply the required gas quantity for nozzle injection. A proportional valve is connected in series after the gas cylinder, which provides feedback on the air pressure signal. This feedback is used to adjust the PWM module in the controller, generating different pulse signals that actuate the solenoid valve for precise control of the driving force. At the executive end of the propulsion branch, the solenoid valve and Laval nozzle are installed, with the Laval nozzle generating the driving force. The bearing branch is responsible for supplying air to the plane air bearing, enabling the device to overcome gravity and achieve floating.

## 3. Kinematics and Dynamics Modeling of the Free-Flying Space Robot

### 3.1. Kinematic Modeling of the FFSR

By neglecting the vertical direction, the motion of the FFSR can be treated as the motion of a rigid body within a plane. Taking into account the motion of the FFSR within this plane, we can establish the reference coordinate system $oX_{f}Y_{f}Z_{f}$ and the body coordinate system $oX_{b}Y_{b}Z_{b}$ for the FFSR. Subsequently, we can derive the transformation matrix from the body coordinate system to the reference coordinate system as

(1) $$R= [\begin{array}{ccc} T_{11} & T_{12} & T_{13}\\ T_{21} & T_{22} & T_{23}\\ T_{31} & T_{32} & T_{33} \end{array}]= [\begin{array}{ccc} c_{\psi }c_{\theta } & -s_{\psi }c_{\phi }+c_{\psi }s_{\theta } & s_{\psi }s_{\phi }+c_{\psi }s_{\theta }c_{\phi }\\ s_{\psi }c_{\theta } & c_{\psi }c_{\phi }+s_{\psi }s_{\theta }s_{\phi } & -c_{\psi }s_{\phi }+s_{\psi }s_{\theta }c_{\phi }\\ -s_{\theta } & c_{\theta }s_{\phi } & c_{\theta }c_{\phi } \end{array}]$$

where *c* and *s* are the cosine and sine, respectively. The Euler angles ψ,θ and ϕ correspond to rotations around the *z*-axis, *y*-axis, and *x*-axis in the reference coordinate system, respectively.

The angular velocities associated with the Euler angles ψ, θ, and ϕ are denoted as $w_{yaw}$, $w_{pitch}$, and $w_{roll}$, respectively. Additionally, the components of the angular velocities of the FFSR in the ontological coordinate system’s *x*-axis, *y*-axis, and *z*-axis are represented as $w_{x}$, $w_{y}$, and $w_{z}$. By considering the relationship between the ontological coordinates and the reference coordinates, we can establish the following equations

(2) $$\begin{array}{cc} w_{x} & =w_{roll}-w_{yaw}\sin\left(\theta \right)\\ w_{y} & =w_{yaw}\cos\left(\theta \right)\sin\left(\phi \right)+w_{pitch}\cos\left(\phi \right)\\ w_{z} & =w_{yaw}\cos\left(\theta \right)\cos\left(\phi \right)-w_{pitch}\sin\left(\phi \right) \end{array}$$

Combining with (1), it can be solved for the Euler angle as

(3) $$\begin{array}{cc} \phi & =\mathrm{atan}\left(\frac{T_{32}}{T_{33}}\right)\\ \theta & =\mathrm{asin}\left(-T_{31}\right)\\ \psi & =\mathrm{atan}\left(\frac{T_{21}}{T_{11}}\right) \end{array}$$

The utilization of Euler angles to represent attitude offers several advantages, including clear physical interpretation, absence of parameter redundancy, and the ability to directly measure the required data using sensors.

### 3.2. Dynamic Modeling of the FFSR

Before proceeding with the dynamics modeling of the FFSR, it is imperative to declare the following assumptions:

**Assumption** **A1.** *The FFSR is treated as a single rigid body, where the influence of elastic deformation is disregarded.*

**Assumption** **A2.** *The space environment is assumed to possess zero gravity.*

**Assumption** **A3.** *The center of mass of the FFSR aligns with its center of geometric symmetry, and the axes of inertia are parallel to the corresponding axes of the body coordinate system.*

According to the kinematic Equation (2), we can obtain

(4) $$\begin{array}{c} \dot{\xi }=R\left(\xi \right)w \end{array}$$

where $\xi ={ [\phi \;\;\theta \;\;\psi ]}^{T}\in R^{3\times 1},\;\;w={ [w_{x}\;\;w_{y}\;\;w_{z}]}^{T}\in R^{3\times 1},\;\;R\left(\xi \right)={ [\begin{array}{ccc} 1 & 0 & -s_{\theta }\\ 0 & c_{\phi } & s_{\phi }c_{\theta }\\ 0 & -s_{\phi } & c_{\phi }c_{\theta } \end{array}]}^{-1}$.

Then, the angular components in the body coordinate system are assumed to be $\vartheta_{x},\;\;\vartheta_{y}$ and $\vartheta_{z}$, and it can obtain

(5) $$\begin{array}{c} w_{x}={\dot{\vartheta }}_{x},\;\;w_{y}={\dot{\vartheta }}_{y},\;\;w_{z}={\dot{\vartheta }}_{z},\;\;{\dot{w}}_{x}={\ddot{\vartheta }}_{x},\;\;{\dot{w}}_{y}={\ddot{\vartheta }}_{y},\;\;{\dot{w}}_{z}={\ddot{\vartheta }}_{z} \end{array}$$

The dynamic equation of attitude motion can be expressed as

(6) $$\begin{array}{c} I\ddot{\vartheta }+\dot{\vartheta }\times \left(I\dot{\vartheta }\right)=\tau \end{array}$$

where $I=\mathrm{diag}\left(I_{x},\;\;I_{y},\;\;I_{z}\right)$, $I_{x},\;\;I_{y}$ and $I_{z}$ are the moments of inertia that the robot rotates around in the three axes of the body coordinate system; $\tau = [T_{x}^{b},\;\;T_{y}^{b},\;\;T_{z}^{b}]$, $T_{x}^{b},\;\;T_{y}^{b}$ and $T_{z}^{b}$ are the torques in the three axes of the body coordinate system, respectively.

The C-W equations can be utilized, along with Euler angles, to depict the attitude of a space robot and to transform reference coordinates to the body coordinate system following the rule of 321. Moreover, these equations can describe the orbital motion of a space robot in an inertial coordinate system when the robot is located near a large spacecraft or a target mission. The orbital dynamics model can be written as

(7) $$\begin{array}{c} \left\{\begin{array}{c} \ddot{x}-2\omega_{0}\dot{y}-3\omega_{0}^{2}x=u_{x}+f_{x}\\ \ddot{y}+2\omega_{0}\dot{x}=u_{y}+f_{y}\\ \ddot{z}+\omega_{0}^{2}z=u_{z}+f_{z} \end{array}\right. \end{array}$$

where $w_{0}$ denotes the desired orbital angular velocity, ${ [x\;\;y\;\;z]}^{T}\in R^{3\times 1},{ [u_{x}\;\;u_{y}\;\;u_{z}]}^{T}\in R^{3\times 1},\;and{ [f_{x},f_{y},f_{z}]}^{T}\in R^{3\times 1}$ represent the position, control force, and external disturbances of the FFSR in each direction within the reference frame system. Then, (7) can be further written as

(8) $$\begin{array}{c} M\ddot{r}+C\dot{r}+\mathrm{Dr}=u+f^{*} \end{array}$$

where $M\in R^{3\times 3}$ denotes the unit matrix, $C= [\begin{array}{ccc} 0 & -2\omega_{0} & 0\\ 2\omega_{0} & 0 & 0\\ 0 & 0 & 0 \end{array}],D= [\begin{array}{ccc} -3\omega_{0}^{2} & 0 & 0\\ 0 & 0 & 0\\ 0 & 0 & \omega_{0}^{2} \end{array}]$, $f^{*}={ [f_{x},\;\;f_{y},\;\;f_{z}]}^{T}$ satisfies $∥\;\;f^{*}∥\leq f_{d}$, $r={ [x,\;\;y,\;\;z]}^{T},\;\;u={ [u_{x},\;\;u_{y},\;\;u_{z}]}^{T}$.

## 4. Design of Fuzzy Sliding Mode Controller

### 4.1. Design of the Sliding Mode Controller

Let $\Lambda_{d}$ and Λ denote the ideal and actual trajectory positions or attitudes of the FFSR, so the tracking error can be expressed as

(9) $$\begin{array}{c} e=\Lambda_{d}-\Lambda \end{array}$$

Define a dynamic sliding mode surface as

(10) $$\begin{array}{c} s_{1}=\dot{e}+\mathrm{ce} \end{array}$$

where $c=\mathrm{diag}\left(c_{x},\;\;c_{y},\;\;c_{z}\right)$ is a positive definite symmetric matrix. Deriving both sides of (10) and taking it into (8) yields

(11) $$\begin{array}{c} {\dot{s}}_{1}=\ddot{e}+c\dot{e}=c\dot{e}+{\ddot{\Lambda }}_{d}-\ddot{\Lambda }=c\dot{e}+{\ddot{\Lambda }}_{d}-M^{-1}\left(u+f^{*}-C\dot{r}+\mathrm{Dr}\right) \end{array}$$

Choosing the exponential convergence law as

(12) $$\begin{array}{c} {\dot{s}}_{1}=-k_{p}s-\varepsilon_{p}\mathrm{sgn}\left(s\right) \end{array}$$

where $k_{p}=\mathrm{diag}\left(k_{x},\;\;k_{y},\;\;k_{z}\right)$ and $\varepsilon_{p}=\mathrm{diag}\left(\varepsilon_{x},\;\;\varepsilon_{y},\;\;\varepsilon_{z}\right)$ denote the positive definite gain matrix, and $\varepsilon_{p}$ satisfies $∥\varepsilon_{p}∥\geq f_{d}$. $\mathrm{sgn}\left(\cdot \right)$ denotes the symbolic function. Then, combining (11) and (12), one can calculate the position tracking control input of the FFSR as

(13) $$\begin{array}{c} u=M\left(k_{p}s+\varepsilon_{p}\mathrm{sgn}\left(s\right)+c\dot{e}+{\ddot{\Lambda }}_{d}\right)+C\dot{r}-\mathrm{Dr} \end{array}$$

Consider a Lyapunov function candidate as

(14) $$\begin{array}{c} V_{1}=\frac{1}{2}s_{1}^{T}s_{1} \end{array}$$

Take the derivative of $V_{1}$ with respect to time yields

(15) $$\begin{array}{c} {\dot{V}}_{1}=s_{1}^{T}{\dot{s}}_{1}=s_{1}^{T}\left(c\dot{e}+{\ddot{\Lambda }}_{d}-M^{-1}\left(u+f^{*}-C\dot{r}+\mathrm{Dr}\right)\right) \end{array}$$

Taking (13) into (15), one can obtain

(16) $$\begin{array}{cc} {\dot{V}}_{1}= & s_{1}^{T}\left(c\dot{e}+{\ddot{\Lambda }}_{d}-M^{-1}\left(M\left(k_{p}s_{1}+\varepsilon_{p}\mathrm{sgn}(s)+c\dot{e}+{\ddot{\Lambda }}_{d}\right)+C\dot{r}-\mathrm{Dr}\right)+f^{*}-C\dot{r}+\mathrm{Dr}\right)\\ = & s_{1}^{T}\left(-k_{p}s_{1}-\varepsilon_{p}\mathrm{sgn}(s)-f^{*}/M\right)\\ = & -k_{p}s_{1}^{T}s_{1}-\varepsilon_{p}∥s_{1}∥-s_{1}^{T}\left(f^{*}/M\right)\\ \leq & -∥k_{p}∥{∥s_{1}∥}^{2}-∥\varepsilon_{p}∥∥s_{1}∥-f_{d}∥s_{1}∥\leq 0 \end{array}$$

It shows that the tracking error can asymptotically converge to zero by choosing appropriate parameters. This completes our proof.

By a similar controller design approach, the design of an attitude tracking controller for FFSR is given as follows. First, the attitude dynamic equation of the FFSR can be expressed as

(17) $$\begin{array}{c} J\dot{w}+w\times \mathrm{Jw}=\tau +\tau^{*} \end{array}$$

where $J_{0},\;\Delta J\in R^{3\times 3}$ is the nominal inertia matrix uncertainty term of the FFSR regarding the propriety coordinate system, and τ and $\tau^{*}$ denote the system’s control torque and the unknown but bounded external disturbance, respectively. Then, considering the sliding mold surface $s_{1}$ defined in (10) and combining it with (17) yields

(18) $$\begin{array}{c} \begin{array}{cc} {\dot{s}}_{1} & =\ddot{e}+c\dot{e}=c\dot{e}+{\ddot{\Lambda }}_{d}-\ddot{\Lambda }\\ \; & =c\dot{e}+{\ddot{\Lambda }}_{d}-{\dot{R}}^{-1}\left(\xi \right)\omega -R^{-1}\left(\xi \right)J_{0}^{-1}\left(-\omega \times J_{0}\omega -\omega \times \Delta J\omega -\Delta J\dot{\omega }+\tau +\tau^{*}\right)\\ \; & =c\dot{e}+{\ddot{\Lambda }}_{d}-{\dot{R}}^{-1}\left(\xi \right)\omega -R^{-1}\left(\xi \right)J_{0}^{-1}\left(-\omega \times J_{0}\omega +\tau +d\right) \end{array} \end{array}$$

where $d=-\omega \times \Delta J\omega -\Delta J\dot{\omega }+\tau^{*}$. Since both the external disturbance and the uncertain inertia matrix of the system are bounded, it can be assumed that $∥R^{-1}\left(\xi \right)J_{0}^{-1}d∥⩽D_{d}$. The exponential convergence law is then chosen as

(19) $$\begin{array}{c} {\dot{s}}_{1}=-k_{a}s_{1}-\varepsilon_{a}\mathrm{sgn}\left(s_{1}\right) \end{array}$$

where $k_{a}=\mathrm{diag}\left(k_{x},\;\;k_{y},\;\;k_{z}\right)$ and $\varepsilon_{a}=\mathrm{diag}\left(\varepsilon_{x},\;\;\varepsilon_{y},\;\;\varepsilon_{z}\right)$ denote the positive definite gain matrix, and $\varepsilon_{a}$ satisfies $∥\varepsilon_{a}∥\geq D_{d}$. According to (18) and (19), it can be calculated that

(20) $$\begin{array}{c} \tau =J_{0}R(\xi ) [\varepsilon_{a}\mathrm{sgn}(s_{2})+k_{a}s_{1}+c\dot{e}+{\ddot{\Lambda }}_{d}-{\dot{R}}^{-1}(\xi )\omega ]+\omega \times J_{0}\omega \end{array}$$

Consider a Lyapunov function candidate as

(21) $$\begin{array}{c} V_{2}=\frac{1}{2}s_{1}^{T}s_{1} \end{array}$$

Taking the derivative of $V_{2}$ with respect to time yields

(22) $$\begin{array}{cc} {\dot{V}}_{2}= & s_{1}^{T}{\dot{s}}_{1}=s_{1}^{T}\left(c\dot{e}+{\ddot{\Lambda }}_{d}-{\dot{R}}^{-1}\left(\xi \right)\omega +R^{-1}\left(\xi \right)J_{0}^{-1}\left(-\omega \times J_{0}\omega +\tau +d\right)\right)\\ = & s_{1}^{T}\left\{\begin{array}{c} c\dot{e}+{\ddot{\Lambda }}_{d}-{\dot{R}}^{-1}\left(\xi \right)\omega \\ +R^{-1}\left(\xi \right)J_{0}^{-1}\left(\begin{array}{c} -\omega \times J_{0}\omega +J_{0}R\left(\xi \right) [\varepsilon_{a}\mathrm{sgn}\left(s\right)+k_{a}s_{1}+c\dot{e}+{\ddot{\Lambda }}_{d}-{\dot{R}}^{-1}\left(\xi \right)\omega ]\\ +\omega \times J_{0}\omega +d \end{array}\right) \end{array}\right\}\\ = & s_{1}^{T}\left(-\varepsilon_{a}\mathrm{sgn}\left(s\right)-k_{a}s_{1}-R^{-1}\left(\xi \right)J_{0}^{-1}d\right)\\ \leq & -∥k_{a}∥{\left\|s\right\|}^{2}-∥\varepsilon_{a}∥\left\|s\right\|-D_{d}\left\|s\right\|\leq 0 \end{array}$$

This demonstrates that by selecting suitable parameters, the tracking error can asymptotically converge to zero. Thus, our proof is concluded.

Although the designed SMCer can be used for position and attitude tracking control of FFSR and weakens the chattering of the system by the designed convergence law, the chattering due to the sign function is still unavoidable. For the designed SMCer, the parameters of the exponential approaching law affect the speed at which the sliding surface is reached and the dynamic quality of system. Therefore, fuzzy control is employed to modify the approaching law parameters, enhancing the system’s ability to fulfill requirements and adapt to uncertain external inputs.

### 4.2. Design of the Fuzzy Controller

Equation (12) can be solved by a first-order linear non-homogeneous differential equation

(23) $$\begin{array}{c} s_{1}\left(t\right)=ce^{-k_{p}t}-\alpha \frac{\varepsilon_{p}}{k_{p}} \end{array}$$

where *c* and α are positive constants. From (23), it can be seen that increasing the value of parameter $k_{p}$ or $\varepsilon_{p}$ can accelerate the approach speed. When the value of $s_{1}$ is large, increasing the value of parameter $k_{p}$ can ensure that the system quickly approaches the sliding mode surface. Nevertheless, from (13), it can be seen that a larger value of parameter $k_{p}$ will also increase the system control input requirements. When the value of $s_{1}$ is small, $\varepsilon_{p}$ plays a major role due to the property of the exponential function. However, when the value of $s_{1}$ approaches zero, the large value of parameter $\varepsilon_{p}$ causes the system chattering. Hence, when the value of $s_{1}$ is large, a smaller value of $k_{p}$ and a larger value of $\varepsilon_{p}$ should be chosen.

**Figure 4:**
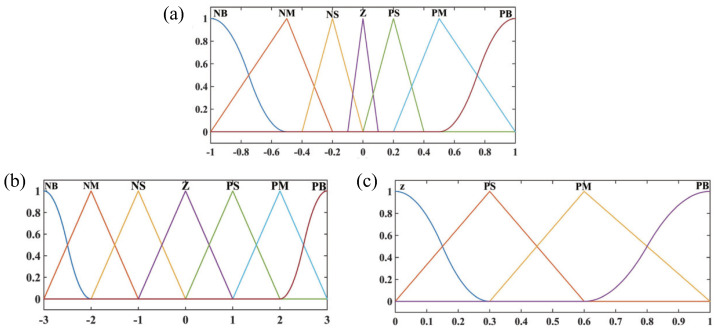
Fuzzy membership function. (**a**) Fuzzy input membership function for *s* and $\dot{s}$. (**b**) Fuzzy output membership function for $\varepsilon_{p}$. (**c**) Fuzzy membership function for $k_{p}$.

Figure 4 shows the designed fuzzy input rule for regulating the parameters $k_{p}$ and $\varepsilon_{p}$. Figure 4a illustrates the fuzzy input rule for *s* and $\dot{s}$. Figure 4b,c illustrate the fuzzy output rule for ∇ε and the fuzzy output rule for $k_{p}$. The fuzzy control rules between *s*, $\dot{s}$ and $k_{p}$ are shown in Table 2, and the fuzzy control rules between *s*, $\dot{s}$ and $k_{p}$ are shown in Table 3.

Considering that the attitude controller (20) of the FFSR operates with the same convergence law and sliding mode surface as the position controller, the fuzzy control rules designed for the latter can also be applied to the attitude controller. This means that the parameters $\varepsilon_{a}$ and $k_{a}$ can be adjusted using the aforementioned fuzzy rules. Therefore, combining the designed fuzzy controller, the position controller (13) and the attitude controller (20) can be rewritten as

(24) $$\begin{array}{c} u=M\left({\hat{k}}_{p}s+{\hat{\varepsilon }}_{p}\mathrm{sgn}\left(s\right)+c\dot{e}+{\ddot{\Lambda }}_{d}\right)+C\dot{r}-\mathrm{Dr} \end{array}$$

(25) $$\begin{array}{c} \tau =J_{0}R(\xi ) [{\hat{\varepsilon }}_{a}\mathrm{sgn}(s_{2})+{\hat{k}}_{a}s_{1}+c\dot{e}+{\ddot{\Lambda }}_{d}-{\dot{R}}^{-1}(\xi )\omega ]+\omega \times J_{0}\omega \end{array}$$

where ${\hat{k}}_{p},\;\;{\hat{\varepsilon }}_{p},\;\;{\hat{k}}_{a},and\;\;{\hat{\varepsilon }}_{a}$ are the gain parameters determined by the fuzzy controller.

### 4.3. Design of the Adaptive Boundary Layer Fuzzy Sliding Mode Control

**Figure 5:**
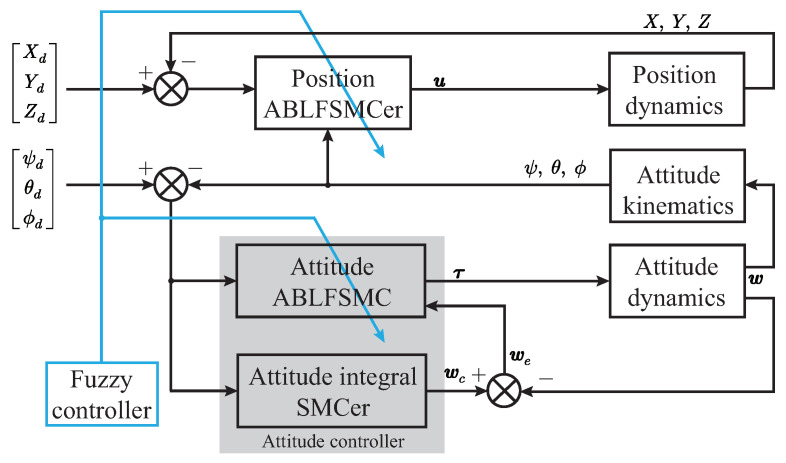
Schematic diagram of the ABLFSMC scheme.

In the context of attitude tracking control for the FFSR, it is not feasible to directly construct the reference angle in the body coordinate system. Instead, a transformation of the angular velocity is necessary. As a result, the design of the attitude tracking controller utilizes a double-closed-loop control method. The outer closed loop adopts the integral SMC to realize the tracking of the Euler angle, and the inner closed loop adopts the exponential approach law SMC to track the angular velocity. Figure 5 shows the schematic diagram of the proposed ABLFSMC scheme.

The outer closed-loop sliding surface of the attitude controller is designed as

(26) $$\begin{array}{c} s_{2}=\xi_{e}+K_{1}\int_{0}^{t}\xi_{e}dt \end{array}$$

where $K_{1}=\mathrm{diag}\left(k_{11},\;\;k_{12},\;\;k_{13}\right)$ denotes the positive definite gain matrix, and $\xi_{e}$ is the error of the Euler angle. An attitude angular velocity $w_{c}$ is constructed as a tracking virtual control term for the angular velocity w of the body, and we have $w_{e}=w_{c}-w$. Consider the virtual control term of the inner loop controller as

(27) $$\begin{array}{c} \omega_{c}=R(\xi )\left({\dot{\xi }}_{c}+K_{1}\xi_{e}\right)+\beta R(\xi )s_{2} \end{array}$$

where β is a defined positive constant.

Consider a Lyapunov function candidate as

(28) $$\begin{array}{c} V_{3}=\frac{1}{2}s_{2}^{T}s_{2} \end{array}$$

Taking the derivative of $V_{3}$ with respect to time yields

(29) $$\begin{array}{c} {\dot{V}}_{3}=s_{2}{\dot{s}}_{2}=s_{2}^{T}\left({\dot{\xi }}_{c}-R^{-1}(\xi )\omega_{c}+R^{-1}(\xi )\omega_{e}+K_{1}\xi_{e}\right) \end{array}$$

By substituting (27) into (29), one has

(30) $$\begin{array}{cc} {\dot{V}}_{3}= & s_{2}^{T}\left({\dot{\xi }}_{c}-R^{-1}(\xi ) [R(\xi )\left({\dot{\xi }}_{c}+K_{1}\xi_{e}\right)+\beta R(\xi )s_{2}]+R^{-1}(\xi )\omega_{e}+K_{1}\xi_{e}\right)\\ = & -\beta \|s_{2}{\|}^{2}+s_{2}^{T}R^{-1}(\xi )\omega_{e} \end{array}$$

It can be seen from (30) that it is necessary to ensure that ${\dot{V}}_{3}\leq 0$, and the error $w_{e}$ should be controlled by the inner loop to be small.

The reference angle $\vartheta_{c}$ for the inner loop controller is determined by integrating $w_{c}$. Subsequently, the inner closed-loop attitude controller mentioned in Section 4.2 is selected. Moreover, to ensure a smooth sliding surface and eliminate chattering resulting from the symbolic function, the symbolic function is replaced with a saturation function, such as

(31) $$\mathrm{sat}\left(s\right)=\left\{\begin{array}{cc} 1,\;\; & s>0.05\\ 20s,\;\; & -0.05<s⩽0.05\\ -1,\;\; & s⩽-0.05 \end{array}\right.$$

Then, the attitude inner loop control SMCer can be obtained as

(32) $$\begin{array}{c} \tau =J_{0} [{\hat{\varepsilon }}_{a}\mathrm{sat}(s)+{\hat{k}}_{a}s+c\dot{e}+{\dot{\omega }}_{c}]+\omega \times J_{0}\omega \end{array}$$

## 5. Simulation and Experimental Results

### 5.1. Simulation Results

In this section, we primarily focus on evaluating the effectiveness of the proposed control scheme by examining the position and attitude tracking performance of the FFSR in the workspace.

The parameters associated with the FFSR are listed in Table 4. The desired tracking trajectory is set to $[\sin\left(0.1t\right),\;\;\sin\left(0.1t+\frac{\pi }{2}\right)-1,\;\;\sin\left(0.1t+\frac{\pi }{2}\right)-1]$, and the desired attitude is set to $[\cos\left(0.1t\right)-1,\;\;\cos\left(0.1t\right)-1,\;\;\cos\left(0.1t\right)-1]$. The initial position and attitude of the system are set to 0. The controller parameters are set to $c=\mathrm{diag}\left(2.5,\;\;2.5,\;\;2.5\right)$, $K_{1}=\mathrm{diag}\left(0.1,\;\;0.1,\;\;0.1\right),\beta =1.5$. The selection of control parameters is primarily based on a trial-and-error method, but they can also adhere to the following principles. A larger gain parameter *c* results in faster convergence of the position error as compared to the velocity error. Similarly, a larger gain parameter $K_{1}$ results in faster convergence of the error but leads to a rapid increase in the control inputs. Additionally, a larger gain parameter β results in faster estimation of the virtual angular velocity but tends to increase the tracking error during the initial tracking phase.

**Figure 6:**
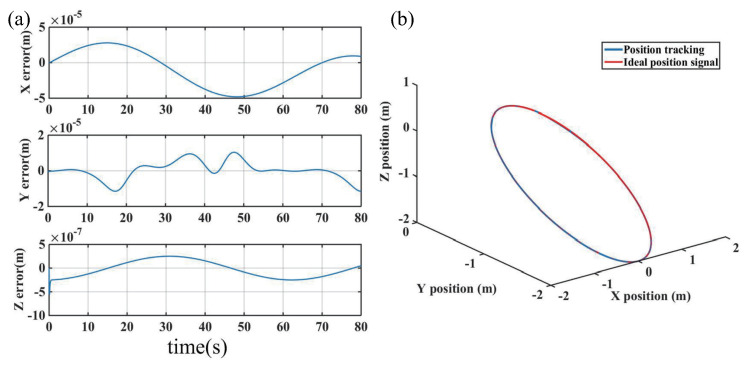
Results of trajectory tracking. (**a**) Tracking 3D space circular trajectory error. (**b**) Three-dimensional (3D) space circular trajectory.

**Figure 7:**
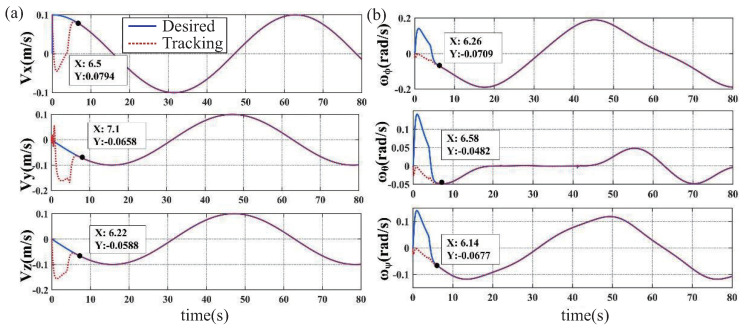
The desired velocity signal and the tracked results. (**a**) Trajectory velocity tracking results. (**b**) Attitude velocity tracking results.

**Figure 8:**
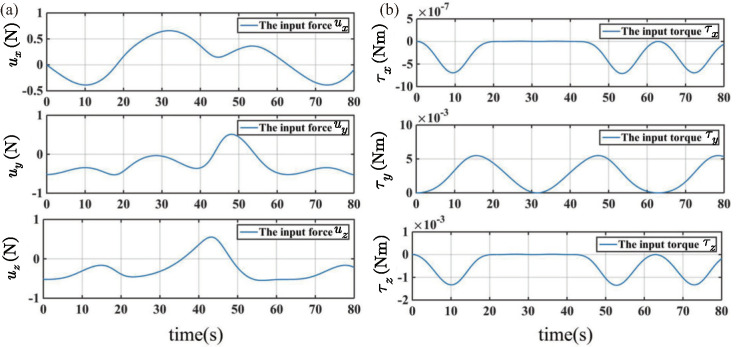
Control input. (**a**) Position control force. (**b**) Attitude control torques.

Simulation results based on the proposed ABFLSMC are presented in Figure 6, Figure 7 and Figure 8. Figure 6a provides insight into the system’s trajectory error when tracking 3D spatial curves in the x,y, and *z* directions. The analysis of trajectory errors reveals that these errors remain within the narrow range of $\pm 5\;\times \;10^{-5}$ m, highlighting the precise tracking capabilities of the proposed control scheme. Furthermore, Figure 6b further supports the efficacy of the control scheme by showcasing the trajectory tracking curves in the workspace. In Figure 7, a depiction of the velocity tracking results of the FFSR demonstrates that with the implementation of the proposed ABLFSMC scheme, the FFSR achieves highly accurate tracking of the desired velocity within a short duration of only 7.1 s. Additionally, Figure 8 provides valuable insights into the control inputs, revealing the successful attainment of smooth control inputs through the utilization of both fuzzy adaptive gain control and the boundary layer technique. The FFSR operates without any contact with the ground, which implies that it is practically unaffected by friction, resulting in a minimal need for propulsive force. As shown in Figure 8, the maximum input force is less than 1 N, making it easily attainable for the jet system employed to propel the FFSR.

### 5.2. Experimental Results

**Figure 9:**
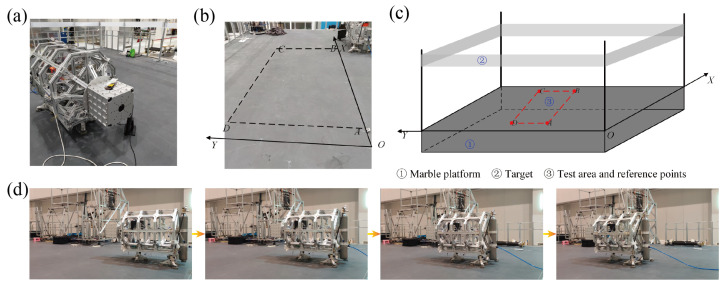
Schematic diagram of the experimental environment. (**a**) FFSR for the trajectory tracking experiment. (**b**) Marble platforms for the tracking experiment. (**c**) Schematic diagram of trajectory tracking. (**d**) The trajectory tracking of the FFSR.

In order to evaluate the motion capability of the proposed controller and the designed FFSR, we conducted tracking control experiments on a smooth marble platform using a rectangular trajectory. The experimental setup is depicted in Figure 9a,b. The coordinates of the four points that define the desired rectangular trajectory are set as follows: *A*(1.5, 2), *B*(5, 2), *C*(5, 4), and *D*(1.5, 4). The FFSR initiates at point *A*, follows the trajectory A→B→C→D→A, and eventually returns to point *A*. In this experiment, we employ a gyroscope and LIDAR to measure the rotational attitude angle and position information. Specifically, the gyroscope is used to determine the angle around the *z*-axis, while the LIDAR and target markers mounted around the marble platform are employed to obtain the position information, as shown in Figure 9c. The movement of the FFSR in performing the trajectory tracking task is shown in Figure 9d.

During the experiment, the control terminal actively transmits precise target position and attitude commands to the system, which are subsequently compared with the highly accurate position and attitude information obtained from the inertial guidance unit and the LIDAR. This meticulous comparison enables us to precisely ascertain the tracking error, which is the discrepancy between the desired and actual position and attitude. Subsequently, the tracking error is seamlessly integrated as an input to the controller, which then robustly computes and generates the required force and torque in each direction to effectively regulate the system’s motion. The signal modulation module, an integral component of the control system, is specifically responsible for generating finely tuned and precisely timed pulse signals, which are diligently transmitted to the dedicated solenoid valves utilized for precise airline control. Ultimately, the activated nozzles, calibrated and synchronized with the pulse signals, providently and consistently deliver thrust in the intended directions, thereby ensuring precise and controlled movement of the system.

**Figure 10:**
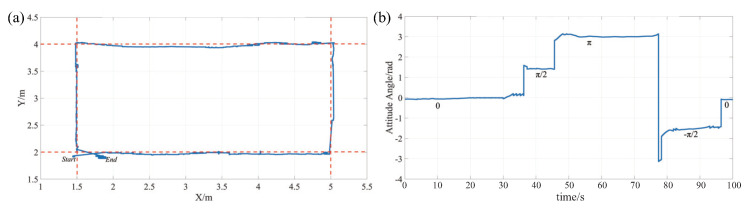
Experimental results of trajectory tracking. (**a**) Diagram of the location of the rectangular trajectory. (**b**) Diagram of rectangular trajectory attitude angle.

Figure 10 visually demonstrates the exceptional capability of the FFSR in closely adhering to the intended rectangular contour as its motion trajectory. Evidently, the FFSR exhibits remarkable precision, with only minor positional and attitude deviations during its movement. Notably, the error values consistently maintain well within the range of 80 mm, effectively meeting the stipulated safe area size requirements throughout the trajectory. These favorable outcomes highlight the commendable performance of the control system in vigorously regulating the FFSR’s trajectory and attaining reliable tracking results. Moreover, in consideration of the potential errors anticipated from the FFSR’s lidar and gyroscope during the acquisition of information, the control system confidently demonstrates its ability to overcome these challenges and achieve exemplary trajectory tracking. These compelling findings reinforce the substantial potential of the FFSR as an invaluable experimental vehicle for the comprehensive ground validation of LSOT-related technologies. However, throughout the experiment, we observed that the proportional valve employed for air pressure regulation demonstrated slow response time and inadequate precision when fine-tuning the air pressure. This limitation significantly contributes to the substantial discrepancies seen in the experimental testing of the developed FFSR when compared to the corresponding simulation results.

## 6. Conclusions

This paper presents the development of a three-degrees-of-freedom FFSR, inspired by water striders, for conducting ground-based validation experiments pertaining to the on-orbit assembly of large-aperture space optical telescopes. The unique gas path design enables a rotational freedom of motion decoupling in the gas drive, thereby facilitating high precision and rapid response motion capability. Additionally, the integration of the intelligent control algorithm fuzzy logic system with the robust control technology SMC effectively enhances the accuracy of trajectory tracking. Simulation and experimental evaluations corroborate the commendable trajectory tracking performance of the proposed controller and the developed FFSR, substantiating its utility as a robust technology verification platform for the ground assembly of space telescopes. Our future efforts will focus on conducting ground-based validation experiments for the on-orbit assembly of space telescopes using the developed FFSR.

## Figures and Tables

**Table 1:** Performance metrics of the designed FFSR.

Parameters	Values
Mass (kg)	158.7
Inertia matrix ($\mathrm{kg}\cdot m^{2}$)	$[\begin{array}{ccc} 25.56 & 0.02 & 3.11\\ 0.019 & 57.26 & 0.04\\ 3.11 & 0.04 & 48.95 \end{array}]$
Geometric dimensions (m)	1.6 × 0.9 × 1
Maximum load (kg)	800
Maximum kinematic thrust (N)	8
Maximum runtime (min)	34.3

**Table 2:** Fuzzy control rules between ∇ε,s and $\dot{s}$.

	$\dot{s}$	NB	NM	NS	Z	PS	PM	PB
*s*								
NB		NB	NB	NM	NM	NS	NS	Z
NM		NB	NM	NM	NS	NS	Z	PS
NS		NM	NM	NS	NS	Z	PS	PS
Z		NM	NS	NS	Z	PS	PS	PM
PS		NS	NS	Z	PS	PS	PM	PM
PM		NS	Z	PS	PS	PM	PM	PB
PB		Z	PS	PS	PM	PM	PB	PB

**Table 3:** Fuzzy control rules between $k_{p},\;\;s$ and $\dot{s}$.

	$\dot{s}$	NB	NM	NS	Z	PS	PM	PB
*s*								
NB		Z	PS	PM	PB	PM	PS	Z
NM		PS	PM	PB	PB	PB	PM	PS
NS		PM	PB	PB	PB	PB	PB	PM
Z		PB	PB	PB	PB	PB	PB	PB
PS		PM	PB	PB	PB	PB	PB	PM
PM		PZ	PM	PB	PB	PB	PM	PZ
PB		Z	PS	PM	PB	PM	PS	Z

**Table 4:** Parameters of the FFSR used for simulation emulation.

Parameters	Values
Mass (kg)	50
Moment of inertia ($\mathrm{kg}\cdot m^{2}$)	diag (8, 11, 11)
Maximum torque ($\tau_{\phi },\tau_{\theta },\tau_{\psi }$) (Nm)	(2, 2, 2.5)
Maximum thrust (N)	4
Disturbance of the position controller (N)	(0.01sin(0.1*t*), 0.02sin(0.1*t*), 0.03sin(0.1*t*))
Disturbance of the attitude controller (N)	(0.1sin(0.1*t*) + 0.1, 0.2sin(0.1*t*) + 0.2, 0.3sin(0.1*t*) + 0.3)

## Data Availability

Data sharing not applicable to this article as no datasets were generated or analyzed during the current study.
